# Randomised Clinical Trial to Analyse the Efficacy of Eggshell Membrane to Improve Joint Functionality in Knee Osteoarthritis

**DOI:** 10.3390/nu14112340

**Published:** 2022-06-03

**Authors:** Fernando Cánovas, María Salud Abellán-Ruíz, Ana María García-Muñoz, Antonio Jesús Luque-Rubia, Desirée Victoria-Montesinos, Silvia Pérez-Piñero, Maravilla Sánchez-Macarro, Francisco Javier López-Román

**Affiliations:** 1 Health Science Department, Campus de los Jerónimos, Universidad Católica San Antonio de Murcia UCAM, 30107 Murcia, Spain; fcanovas@ucam.edu (F.C.); msabellan@ucam.edu (M.S.A.-R.); amgarcia13@ucam.edu (A.M.G.-M.); ajluque@ucam.edu (A.J.L.-R.); dvictoria@ucam.edu (D.V.-M.); sperez2@ucam.edu (S.P.-P.); msanchez4@ucam.edu (M.S.-M.); 2 Primary Care Research Group, Biomedical Research Institute of Murcia (IMIB-Arrixaca), 30120 Murcia, Spain

**Keywords:** knee pain, dietary supplement, stiffness, glycosaminoglycans

## Abstract

Osteoarthritis is a source of chronic pain and disability. Dietary supplements have been shown to be a more secure option than NSAIDS. Particularly, the eggshell membrane has demonstrated efficacy in relieving joint pain and stiffness. A clinical trial was designed in which three groups were assigned to two different doses of this supplement and compared to a placebo control group. The primary outcome variable was knee pain, which was assessed using a visual analogue scale. Secondary outcome variables were knee functional ability, quadriceps muscle strength (assessed by isometric and isokinetic trials), and quality of sleep. All groups showed a significant decrease in pain perception, although maximum values were obtained in the high-dose group. Isokinetic and isometric trials showed a significant increase in strength in the high-dose group. Eggshell membrane showed the potential to reduce pain and stiffness symptomatology. Here, for the first time, two quantitative variables (mobility and strength of knee joint) were used to accurately evaluate changes in the quality of life of subjects affected by knee joint pain. The results of this study indicate a dose-dependent response, which should be taken into account for later use in therapeutics to establish the correct dosage.

## 1. Introduction

Increasing life expectancy has led to structural changes in current human populations [1]. Ageing is posing a challenge for increasing care needs due to physical and mental multimorbidities in the 21st century [2] since evidence that longevity coincides with an extended period of good health is scarce [3]. In fact, ageing and increased body mass index (BMI) are leading causes of osteoarthritis (OA) [4].

OA is the most prevalent joint disorder and source of chronic pain and disability in developed countries [5,6]. This degenerative disease of the joint affects the locomotor system and is characterised by a loss of articular cartilage, as well as an osseocartilaginous proliferation of the subchondral and articular margins [7]. About 10% of adult people suffer some type of moderate to severe OA, and this percentage increases with age and is even more accentuated in those over 50 to 55 years old [8]. The hips and knees are the joints most affected by this disorder.

Acute pain is a commonly associated symptom; therefore, pharmacotherapy is indicated as treatment through the use of analgesics and non-steroidal, anti-inflammatory drugs (NSAIDS) [7,9]. However, most of these treatments have shown limited effectiveness and have induced several multiorgan toxicities [10]. Currently, dietary supplements are commonly used to counteract pain in a combination of hyaluronic acid, glucosamine, and chondroitin. However, some dietary supplements have also been examined [11,12] and have been shown to be a more secure option than NSAIDS [13].

Among them, eggshell membrane has demonstrated efficacy in relieving joint pain and stiffness [14,15,16,17,18,19]. The eggshell membrane is composed mainly of fibrous collagen proteins, types I, V, and X. It also contains glycosaminoglycans, such as chondroitin sulphate and dermatan sulphate, and hexosamines, such as glucosamine [20]. In addition, hyaluronic acid has been shown to be present in significant amounts [21]. Therefore, ESM has been evaluated as a possible treatment for OA as a natural source of an optimal combination of such compounds [17,19]. Despite the fact that some clinical trials have demonstrated promising results in improving the functionality of the knee joint [19], some lack of knowledge still remains regarding dosage.

Therefore, the objective of this study was to determine the efficacy of a food supplement extracted from the internal membrane of the eggshell on joint functionality (knee functional ability and quadriceps muscle strength) and perceived pain (visual analogic scale and quality of sleep) in individuals diagnosed with OA, after a consumption period of eight weeks. Two doses were tested in order to evaluate efficacy in a placebo-controlled clinical trial.

## 2. Materials and Methods

**Figure 1:**
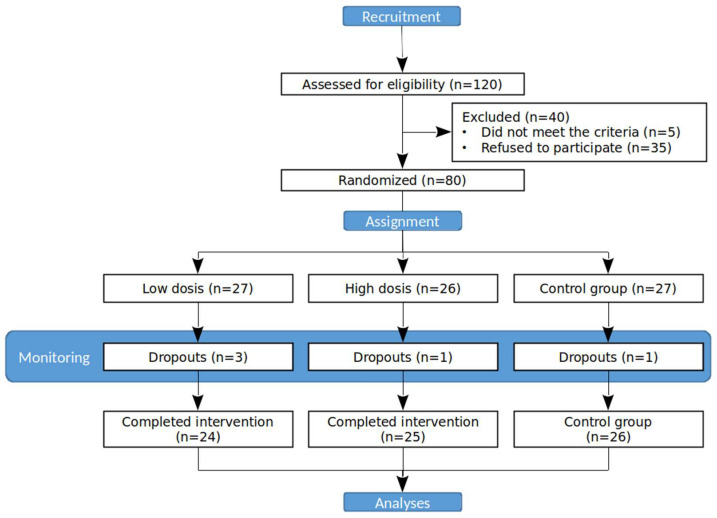
Flowchart representing the different stages of the trial. Randomisation profile shows flow of patients in the three groups: high-dose (500 mg), low-dose (300 mg), and control group.

A randomised, controlled, double-blind, single-centre clinical trial was designed, in which three groups were assigned to different doses of an internal membrane, eggshell-based supplement (Figure 1). This study was conducted in 2018–2019 on 80 patients over 18 years of age with diagnosed knee osteoarthritis and chronic knee pain.

The trial design followed CONSORT guidelines. Written, informed consent was signed by all participants after approval by the University’s Institutional Science Ethics Committee. Inclusion criteria were as follows: subjects diagnosed with arthrosis with functional grades I–III according to criteria from the American College of Rheumatology, Atlanta; persistent knee pain associated with this pathology, with initial punctuation of at least 30 mm in a visual analogue scale of 10 cm; and absence of chronic treatment with narcotics, NSAIDs, or immunosuppressants. Exclusion criteria were as follows: terminal disease; known chronic inflammatory diseases affecting the musculoskeletal system (rheumatoid arthritis, gout, pseudo-gout, Paget’s disease, chronic pain syndrome, etc.); other serious illnesses that would limit the execution of aerobic or resistance exercises (musculoskeletal conditions, limiting pneumopathy, presence of arrhythmia); body mass index (BMI) above 32; subjects who, at the time of the study, were being treated with glucosamine, chondroitin sulphate, collagen, or hyaluronic acid infiltrations, or consuming any supplement indicated for joint health; subjects under pharmacotherapy (narcotic drugs, steroid anti-inflammatory drugs, or immunosuppressants); known allergy to eggs; pregnant or breastfeeding women; and incapacity to understand informed consent.

Treatment consisted of once-daily oral ingestion of 300 mg (low dose) and 500 mg (high dose) of ESM^®^ eggshell membrane (Torolis Explotaciones, S.L., Navarra, Spain) in vegetarian capsules that were stored in closed containers at ambient temperature. The control group consumed a placebo based on encapsulated maltodextrin matching the weight and aspect of treatment. Eggshell membrane extract was composed mainly of proteins (>90%), fibrous collagen types I, V, and X (<13%), and elastin (<4–5%). The extract also contained glycosaminoglycans, such as chondroitin sulphate (<2%); dermatan and keratin sulphates (<1%); and hexosamines, such as glucosamine (<2%), hyaluronic acid (<2%), IGF-1 (12 ng/g), TGF-beta (0.75–7.23 ng/g), calcitonin (10–25 ng/g), and progesterone (0.3–0.33 ng/g). Clinic visits were scheduled for subjects at study initiation and at the end of 8 weeks following the onset of treatment. Compliance with the treatment was evaluated during the last visit via patient interview and by counting the number of unused capsules still remaining. Lifestyle variables included a dietary recall interview of the previous 3 days before starting the nutritional supplement and the last 72 h before the end of the 8-week consumption of the product. The level of physical activity was recorded using the Global Physical Activity Questionnaire (GPAQ), and results were expressed as MET-min/week. Specific rules were given to subjects about maintaining the same level of physical exercise during the trial. Weight was registered via bioimpedance.

The primary outcome variable was knee pain which was assessed using a visual analogue scale (VAS) of 10 cm, defining extreme limits such as ‘no pain at all’ and ‘pain as bad as it could be’ [22]. Values lower than 4 in VAS meant mild to moderate pain, a value between 4 and 6 implied the presence of moderate to severe pain, and pain with values higher than 6 implied the presence of very intense pain [22]. This scale was recorded before and after strength assessment at both baseline and final visits. VAS scale was also recorded throughout the 8 weeks at the time the participant awoke each morning through the completion of a diary. Adverse events were also recorded.

Secondary outcome variables were knee functional ability, quadriceps muscle strength (assessed by isometric and isokinetic trials), and quality of sleep.

Functional ability was evaluated using the Western Ontario and McMaster Universities Osteoarthritis (WOMAC) index, through an adapted Spanish version of the WOMAC questionnaire [23]. This questionnaire determined pain and functional ability. Within the questionnaire, there are three subscales, 5 questions referring to joint pain, 2 questions on stiffness, and 17 on functional capacity. Each question is graded from 0 to 4, with 0 meaning ‘none’ and 4 meaning ‘very much’. The WOMAC questionnaire had to be completed before each of the strength assessments at both visits.

Muscle strength was determined using a Biodex System 3 Dynamometer (Biodex Medical System, Shirley, New York, NY, USA) in order to assess isometric (straining your muscles without moving or bending your joints) and isokinetic (performed at a consistent speed, which can be increased as you progress) strength of the knee flexors and extensors. It is important to note that changes in strength should be associated with changes in perceived pain and not with changes in the physical condition of the muscle. Trials involved two maximum isokinetic and two continuous maximum isometric repetitions at 60°/s and 90°/s on their right leg, respectively. Isokinetic trials collected information about muscle peak torque (PT, i.e., the maximum force that a muscle group can produce); total work of the maximum repetition (TWMR, i.e., maximum strength exerted at the time of making the movement); and total work (TW, i.e., strength exerted during all repetitions to cause the displacement of the leg and overcome the resistance offered by the isokinetic dynamometer protocol). Isometric trials assessed muscle peak torque (PT/BW) and maximum average peak torque (MAPT, i.e., medium torque). Verbal encouragement was provided during the tests, and adequate rest and recovery times were provided between contractions in order to minimise fatigue.

Quality of sleep was assessed by the Pittsburgh Sleep Quality Index (PSQI) [24], which has shown good psychometric properties and validity for application in the adult population. PSQI evaluated seven domains: subjective sleep quality, sleep latency, sleep duration, habitual sleep efficiency, sleep disturbances, use of sleeping medication, and daytime dysfunction over the past month. Each subject self-rated each of these seven areas of sleep. Each domain is scored based on a 0–3 on the Likert scale: 0 (very good), 1 (good), 2 (poor), and 3 (very bad). The PSQI score was calculated as the sum of the scores, which varied between 0 and 21. This test was filled in before each of the strength evaluations at each visit.

ANOVA with repeated measures was used to compare variables that were obtained at the beginning and end of this trial. All variables were checked for normality. Homogeneity of variables among groups was also checked in the baseline in order to avoid confounding variables. Bonferroni correction was used for all comparisons between intervention groups and control. Type I error rate was set at α = 0.05. Analyses were performed by using SPSS statistical software v.21.0 (IBM Corp., Armonk, NY, USA, EEUU).

## 3. Results

A total of 80 out of 120 subjects assessed for eligibility were randomised (see Figure 1 for a trial flow), from which 75 (93.75%) completed this study (36 men and 39 women, mean age 38.40 ± 13.54 years old). Five individuals did not match the criteria for inclusion in the trial, and thirty-five individuals refused to participate. Demography information is provided for each group in Table 1. The placebo group included a total of 26 participants with a mean age of 41 ± 14.36 years old (10 men and 16 women). The low-dose group showed an average age of 37.38% ± 12.29 years old (12 men and 12 women). The high-dose group had an average age of 36.36 ± 13.54 years old (14 men and 11 women). At the beginning of this study, the percentage of body fat in the control group was 27.75 ± 1.7%, while for experimental groups, it was 26.89 ± 1.7% (300 mg) and 24.39 ± 1.7% (500 mg). By the end, the control group registered a body fat percentage of 28.32 ± 1.7%, while the 300 mg and 500 mg consumption groups registered a body fat percentage of 25.98 ± 1.7% and 24.82 ± 1.7%, respectively. No significant differences were found. BMI (normal vs. overweight/obesity) did not show significant differences between groups, as well as between initial and final conditions after ending of this study.

At the beginning of this study, subjects indicated self-medication with NSAIDs in 18.5%, 14.8%, and 19.2% of control, low-, and high-dose experimental groups, respectively. Although subjects did not report changes in such medication, diary annotations recorded punctual pain medication in 25% of them.

All three groups showed a statistically significant decrease in pain perception by the end of this study (Table 2). Furthermore, there were significant differences between groups, as the high-dose group showed a statistically significant reduction in pain with respect to the control group (Table 2).

The evolution of pain perception as soon as a subject awoke during the study period can be also followed in Table 3. No differences were observed between groups in the baseline (*p* > 0.1), registering 4.75 ± 1.16 points (control), 4.69 ± 1.18 points (low dose), and 5.23 ± 1.32 points (high dose). A progressive decrease in the evolution of pain can be observed for all groups, and the lowest values on the scale were achieved in the last week for all groups (Table 3). The control group showed mild to moderate perceived pain by the end of this study (3.91 ± 2.16 points), although this decrease (1.27 points) was not significant (*p* > 0.05). The other two groups registered a significant decrease in perceived pain, decreasing to 3.13 ± 1.43 points (mild to moderate pain) in the low-dose group, and decreasing to 2.98 ± 1.51 points (mild pain) in the high-dose group (Table 3). Comparison of the evolution among the three study groups showed significant differences, and the high-dose group significantly reduced perceived pain with respect to the control group (*p* < 0.014).

The WOMAC results were homogeneous among the three groups at the beginning of this trial (Table 2). The control group started this study with values of 25.96 ± 2.7 points, while the low- and high-dose groups started with similar values of 26.29 ± 2.7 and 24.80 ± 2.7 points, respectively (*p* > 0.1). Despite the fact that all three groups showed a significant decrease in the WOMAC scale score (Table 2), both groups that consumed this product showed a more pronounced decrease, and therefore an improvement in functional capacity and quality of life (Table 2).

Isokinetic and isometric trials showed no significant differences at the beginning of this study for all variables included (Table 4). Although an increase in strength, measured as PT, TWMR, and TW, with respect to baseline values, was observed in those groups subjected to consumption of eggshell membrane product at both doses, the decrease in pain was only significant in the high-dose group for all the variables studied here, accounting for 7.2, 10.04 and 47.42 N × m, respectively (Table 4). The control group registered a decrease in strength for two variables (PT = −1.48 N x m and TW = −3.21 N × m), although such results lacked statistical significance (Table 3). Similar results were found for isometric trials. The high-dose group showed a significant increase in strength for both PT (25.18 N × m) and MAPT (20.76 N × m). The low-dose group showed an increase in PT (20.36 N × m) and MAPT (16.29 N × m), although the latter lacked statistical significance (Table 4). PT registered non-significant results for the control group that showed a slight decrease (−0.41 N × m), and MAPT registered a slight increase that also lacked significance (1.01 N × m).

Pittsburgh test scores provided similar scores at the beginning of this trial for all three groups (Table 2), and no significant differences were found between them. At the end of the study, the control group kept the same scores. Both treated groups showed a decrease, though this was higher in the high-dose group (1.6 points) than in the low-dose group (0.46 points). These differences observed in the high-dose group were significantly different from those obtained for the other two groups, indicating an improvement in the quality of sleep for the high-dose group compared with the other two groups.

Subjects did not report any adverse events related to the consumption of the product administered.

## 4. Discussion

Pain due to osteoarthritis is known to induce long-term consequences on health as a consequence of a lack of exercise [16]. In fact, other comorbidities, such as being overweight or ageing, could aggravate these consequences and could influence other ones as a result of immobility due to pain.

Eggshell membrane showed the potential to reduce such symptomatology. Although all of the groups registered a decrease in perceived pain, both doses used in this study induced a bigger reduction than in the control group, indicating a relationship between eggshell membrane consumption and reduction in pain. Such results are compatible with previous studies that demonstrated the efficacy of this nutraceutical to alleviate knee pain, symptomatology, and joint functionality [14,15,16,17,18,19]. These results are higher than those registered for other supplements such as curcuma [12]. However, dose-dependent efficacy was registered in our study (Table 2). Despite the fact that all groups showed a reduction in pain, objective measures of improvement in functional capacity and sleep quality support such a hypothesis. A 500 mg dose induced a reduction in perceived pain of 1.1 points more than a 300 mg dose. Furthermore, a slight reduction in functional capacity and an improvement in sleep quality were also registered for high vs. low doses. All three signs together indicate an improvement in knee pain, although an increase in the dose used does not show a proportional effect in these variables.

Reduction in perceived pain was decremental and was observed during the entire study period, as registered by the information collected as soon as the subject awoke in the morning. Such a variable registered a significant weekly evolution of pain through the study for the high dose, showing a final reduction of 0.69 points more than the low dose (Table 3). Those results are also in agreement with the trials performed in this study. The WOMAC scale score and quality of sleep index (Table 2) showed a decrease in both groups of consumers, therefore registering an improvement in functional capacity and quality of life, although differences between doses were not significant. This is probably due to the fact that a decrease in pain and a recovery of knee joint functionality perception could make those two variables (functional capacity and quality of life) improve but only up to a certain level due to the subjectivity of tests. Perception of improvement could probably be achieved in both dosages, only showing slight differences between dosages registered by the tests used.

In fact, such an observation could be in agreement with the results obtained for muscle strength. All variables studied here registered an increase in both consumption groups, although a higher dose-dependent effect was registered than for other tests (Table 4). Isokinetic trial variables at 60°/s (PT, TWMR, and TW) show the highest differences: between four and ten times greater for the higher dose. Isometric trials assessed at 90°/s (PT and MAPT) also show significant differences between control and treatment groups, but with fewer differences between the low and high doses. These results show a significant improvement in muscle strength as a result of a reduction in pain perception. Moreover, such an effect is dose-dependent. Such a conclusion can make a difference when applying the use of eggshell membranes in therapeutics regarding knee pain; therefore, perceived pain reduction and functionality increase do not entirely reflect all of the benefits of consuming eggshell membranes. This could probably be due to the objectivity of muscle strength assessment through trials compared with pain and functionality tests, which would indicate a physiological improvement and, therefore, a more objective observation about the outcome of the use of eggshell membranes to treat knee pain.

Although previous studies addressed the study of the efficacy of eggshell membrane as a supplement to alleviate knee joint pain [14,15,16,17,18], here, for the first time, two quantitative variables (mobility and strength of knee joint), as well as qualitative ones (pain, functional ability, and sleep quality), were monitored to accurately evaluate changes in the quality of life of subjects affected by this common pathology. Such a variable set extends the findings of previous studies that have already observed changes in the functionality of knee joints by measuring the range of moti- on [19]. This study reports an improvement in all of the variables monitored in those subjects who were consuming eggshell membrane, most likely resulting from physiological changes in the musculature associated with the amelioration of knee functionality and a better quality of life due to a reduction in perceived pain. Moreover, our results also indicate a dose-dependent response, which should be taken into account for later use in therapeutics to establish the correct dosage.

## Figures and Tables

**Table 1:** Demography characteristics of the 75 subjects who finished this study by group.

	Control (*n* = 26)	Exp 300 (*n* = 24)	Exp 500 (*n* = 25)	Total
**Age (mean ± SD)**	41.31 ± 14.36	37.38 ± 12.29	36.36 ± 13.54	38.44 ± 13.54
**BMI (mean ± SD)**	25.3 ± 4.0	25.4 ± 4.0	24.6 ± 3.0	25.1 ± 3.6
BMI < 25; *n* (%)	12 (46.2%)	10 (41.7%)	15 (60%)	37 (49.3%)
BMI ≥ 25; *n* (%)	14 (53.8%)	14 (58.3%)	10 (40%)	38 (50.7%)
**Sex; *n* (%)**				
Women	16 (61.5%)	12 (50.0%)	11 (44.0%)	39 (52.0%)
Men	10 (38.5%)	12 (50%)	14 (56.0%)	36 (48.0%)

**Table 2:** Visual analogue scale (VAS) for perceived pain, functional capacity by means of the WOMAC test, and Pittsburgh Sleep Quality Index (PSQI) for each of the visits and doses. Δ represents the increment from the start to the end of this trial. Significance level for the differences between values at the beginning and end of this trial.

		Control	Low Dose	High Dose
**VAS (*p* = 0.001)**	**Initial**	4.90 (1.48)	4.67 (1.37)	5.42 (1.48)
	**Final**	3.64 (1.40) ***	2.25 (1.66) ***	1.90 (1.78) ***
	**Δ**	−1.269	−2.417	−3.52
**WOMAC (*p* = 0.183)**	**Initial**	25.96 (13.42)	26.29 (15.81)	24.80 (10.92)
	**Final**	20.32 (13.17) **	16.04 (12.28) ***	14.52 (10.20) ***
	**Δ**	−5.64	−10.25	−10.28
**PSQI (*p* = 0.301)**	**Initial**	6.77 (2.82)	6.00 (2.34)	6.60 (3.76)
	**Final**	6.27 (3.14)	5.54 (2.89)	5.00 (3.03) **
	**Δ**	−0.5	−0.46	−1.6

Significance levels: *** < 0.001 < ** < 0.01 < * < 0.05.

**Table 3:** Weekly mean (standard deviation) of visual analogue scale (VAS) for perceived pain during the period of study. Δ represents the increment from the start to the end of this trial. Significance level for the differences between values at the beginning and end of this trial.

	Week1	Week2	Week3	Week4	Week5	Week6	Week7	Week8	Week9	Δ
**Control**	4.75 (1.16)	4.74 (1.72)	4.62 (1.93)	4.52 (2.27)	4.43 (1.88)	4.31 (2.27)	4.13 (2.21)	4.19 (2.29)	3.91 (2.16)	−0.835
**Low dose**	4.69 (1.18)	4.37 (1.45)	3.99 (1.47)	3.77 (1.41)	3.89 (1.17)	3.53 (1.41)	3.62 (1.42)	3.32 (1.57)	3.13 (1.43)	−1.559
**High dose**	5.23 (1.32)	4.93 (1.55)	4.3 (1.74)	4.08 (1.7)	3.84 (1.68)	3.65 (1.81)	3.4 (1.71)	3.23 (1.59)	2.98 (1.51)	−2.250 ***

Significance levels: *** < 0.001 < ** < 0.01 < * < 0.05.

**Table 4:** Isokinetic trials variables at 60°/s: muscle peak torque (PT), total work of the maximum repetition (TWMR), and total work (TW). Isometric trials assessed at 90°/s: muscle peak torque (PT) and maximum average peak torque (MAPT). Δ represents the increment from the start to the end of this trial. Significance level for the differences between values at the beginning and end of this trial. *p* values indicate significance level for comparison of groups by the duration of this trial for each variable. Units were measured in N × m.

Isokinetic at 60°/s				
		Control	Low Dose	High Dose
**PT (*p* = 0.048)**	**Initial**	57.93 (22.36)	62.15 (24.28)	59.28 (26.52)
	**Final**	56.45 (22.66)	62.98 (24.92)	66.48 (28.23)
	**Δ**	−1.48	0.83	7.2 ***
**TWMR (*p* = 0.016)**	**Initial**	64.60 (28.48)	68.91 (30.40)	66.89 (31.94)
	**Final**	64.66 (27.73)	70.53 (30.17)	76.93 (35.08)
	**Δ**	0.06	1.62	10.04 ***
**TW (*p* = 0.017)**	**Initial**	294.89 (137.08)	309.38 (150.72)	297.72 (147.07)
	**Final**	291.68 (130.01)	322.61 (144.70)	345.13 (167.64)
	**Δ**	−3.21	13.23	47.42 ***
**Isometric at 90°/s**				
		**Control**	**Low Dose**	**High Dose**
**PT (*p* = 0.018)**	**Initial**	145.41 (61.07)	137.99 (61.16)	146.73 (70.07)
	**Final**	145.00 (61.42)	158.35 (56.72)	171.92 (84.64)
	**Δ**	−0.41	20.36 **	25.18 ***
**MAPT (*p* = 0.016)**	**Initial**	138.16 (58.35)	137.40 (52.22)	141.27 (57.34)
	**Final**	139.17 (59.23)	153.69 (52.52)	161.04 (78.39)
	**Δ**	1.01	16.29	20.76 ***

Significance levels: *** < 0.001 < ** < 0.01 < * < 0.05.

## Data Availability

Not applicable.
